# Kinetics of Zinc Corrosion in Concrete as a Function of Water and Oxygen Availability

**DOI:** 10.3390/ma12172786

**Published:** 2019-08-29

**Authors:** Petr Pokorný, Milan Kouřil, Vojtěch Kučera

**Affiliations:** 1 Klokner Institute, Czech Technical University in Prague, 166 08 Prague, Czech Republic; 2 Institute of Metals and Corrosion Engineering, University of Chemistry and Technology in Prague, 166 28 Prague, Czech Republic

**Keywords:** corrosion of steel in concrete, hot-dip galvanized reinforcement, zinc, kinetics, electrical resistance, calcium hydroxyzincate (CHZ)

## Abstract

This paper studies the effect of water as an oxidation agent and also of oxygen on zinc corrosion kinetics in active state in concrete, using high-sensitivity electrical resistance sensors. It was proven that zinc corrosion in active state is strongly affected by the presence of water at its surface. Zinc corrosion in real concrete in the absence of water can be misinterpreted as salt passivity. The presence of oxygen results in an increase of zinc corrosion rate, however at pH 12.6, passivity can occur. It was verified that corrosion products consisting primarily of Ca[Zn(OH)_3_]_2_·2H_2_O cannot effectively passivate zinc surface in concrete, even after 1800 h of exposure and zinc, or hot-dip galvanized steel can corrode at an unacceptable corrosion rate (more than 4 µm·a^−1^).

## 1. Introduction

Reinforcement with steel profiles (rods, rebars, braidings) is a process necessary to ensure sufficient tensile strength of concrete. Reinforced concrete provides a combination of properties, making it one of the core materials used in civil engineering [1,2].

On the other hand, corrosion damage to this reinforcement is what limits longevity of constructions from reinforced concrete. Steel in concrete corrodes in passive state with an acceptable, that is, a very low corrosion rate (r_corr_ < 0.1 µm·a^−1^). This corresponds to the formation of a very thin passive layer from amorphous hydrated iron oxides (only a few monolayers) [2,3]. Activation of the surface of the reinforcement (rebar) is tied to pH reduction of the concrete covering layer, due to interaction with atmospheric CO_2_ (very slow process) [4]. An unacceptable rate of corrosion (r_corr_ > 2.0 µm·a^−1^) is usually caused by contamination of the covering layer by chlorides (from deicing salts or from aerosol in coastal areas) [2,3].

A protective layer on rebar created by conventional hot-dip galvanizing has several indisputable advantages—primarily, it provides high resistance for the coating, even in carbonated concrete, higher tolerance to chlorides, and also its affordable price [5]. Research [6] as well as practice [7] showed that significant corrosion damage (with hydrogen evolution—see Equation (1)) to the coating in fresh and curing concrete increases porosity at the interface with the cement paste [6,8,9], reducing bond strength between the rebar and concrete.

(1) $$2H_{2}O+2e^{-}\to H_{2}+2{\mathrm{OH}}^{-}$$

Behavior of hot-dip galvanized rebar in concrete is a matter of discussion. Original works on this topic emerged in the 1980s, and they describe passivation of the galvanized steel surface in a model pore solution of pH < 13.3 [10,11,12,13]. Protective properties are ascribed to the crystalline corrosion product—Ca[Zn(OH)_3_]_2_·2H_2_O (calcium hydroxyzincate—CHZ) [11,14,15]. Its formation is described by Equation (2) [5,11]:

(2) $$2\mathrm{Zn}{\left(\mathrm{OH}\right)}_{2}+2H_{2}O+\mathrm{Ca}{\left(\mathrm{OH}\right)}_{2}\to \mathrm{Ca}{\left[\mathrm{Zn}{\left(\mathrm{OH}\right)}_{3}\right]}_{2}\cdot 2H_{2}O$$

Using current measurement techniques, the efficient passivation capability was challenged [16,17,18]. One alternative theory suggests that the presence of Ca^2+^ cations in model pore solution causes destabilization of the original amorphous layer of ZnO and Zn(OH)_2_, described in detail in alkaline solutions of similar pH (12.6–12.8); however, without Ca^2+^ [19,20]. Formations of ZnO and Zn(OH)_2_ are described in Equations (3) and (4) [19]:

(3) $$\mathrm{Zn}+2{\mathrm{OH}}^{-}\to \mathrm{ZnO}+H_{2}O+2e^{-}$$

(4) $$\begin{array}{c} \mathrm{ZnO}+H_{2}O+2{\mathrm{OH}}^{-}\to {\left[\mathrm{Zn}{\left(\mathrm{OH}\right)}_{4}\right]}^{2-}\\ {\left[\mathrm{Zn}{\left(\mathrm{OH}\right)}_{4}\right]}^{2-}\to \mathrm{Zn}{\left(\mathrm{OH}\right)}_{2}+2{\mathrm{OH}}^{-} \end{array}$$

Nevertheless, all above referenced measurements were realized in model pore solution and not in real construction grade concrete. Therefore, their conclusions cannot be used for the description of zinc (or galvanized steel) corrosion kinetics in real concrete, since their results do not account for crucial development stages of the environment (wetting and drying cycles of concrete, continuous release of Ca^2+^ cations, limitation by and rate of transport of atmospheric oxygen, etc.).

The goal of this paper is the precise description of zinc corrosion kinetics in real concrete. This research sets itself apart by using high-sensitivity resistance electrical sensors for the direct and precise measurement of corrosion rate, unlike previous papers, which calculated corrosion indirectly. The fundamental impact of this work is establishing direct passivation efficiency of a layer of zinc corrosion products precipitated in concrete, based on the aforementioned Ca[Zn(OH)_3_]_2_·2H_2_O (dominant constituent), and the effect of increased oxygenation of concrete on passivation of zinc (facile formation of ZnO and Zn(OH)_2_).

These results significantly affect prediction of the longevity and evolution of mechanical properties of reinforced concrete constructions. 

## 2. Materials and Methods

**Figure 1:**
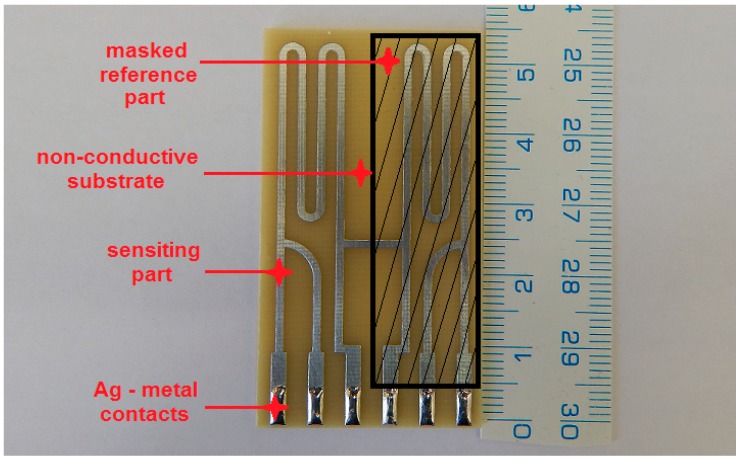
High-sensitivity electrical resistance sensor made of 50 µm zinc foil.

In this experimental program, high-sensitivity electrical resistance sensors were used to measure actual corrosion rates in relation to changes in relative humidity (% RH). Zinc foil had guaranteed purity of 99.999%. High-sensitivity electrical resistance sensors consist of a reference and sensing part (Figure 1). The reference part compensates for the changes of electrical resistance caused by changes in temperature and is always covered with epoxy glue. Reduction of thickness due to corrosion on the sensing part was measured by a 4-wire connection using the ACD-03 datalogger (MetriCorr, Rødovre, Denmark). The technique is described in detail, along with the current generation of high-sensitivity electrical sensors, in other papers [21].

**Figure 2:**
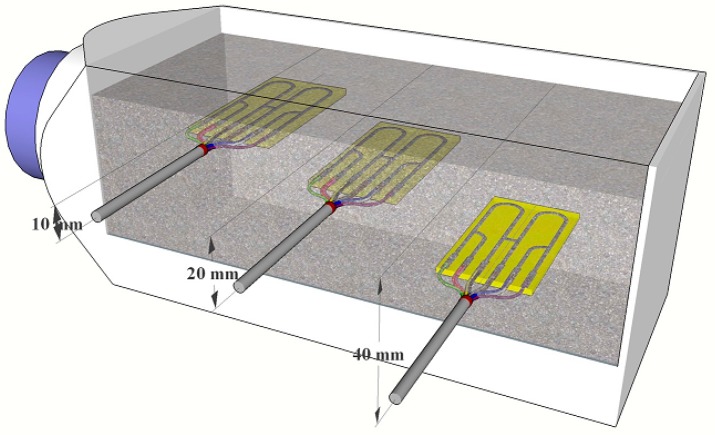
Schematic of placement of individual high-sensitivity electrical resistance sensors in the concrete sample with different distances to the surface.

For each concrete sample, three high-sensitivity electrical resistance sensors, placed in different distances, were used (the distances are shown in Figure 2). To prepare concrete samples, prism containers (molds) placed on wooden legs were used (glued with epoxy). A metallic track of the individual high-sensitivity electrical resistance sensors was softly abraded (P1200) and cleaned by ethanol.

A commercial concrete mixture C16/20 was used in all experiments. Cement composition was guaranteed by its producer (see Table 1). A small portion of the mixture was sifted, and the acquired cement sample was analyzed regarding the concentration of CrO_4_^2−^ and Cl^−^, which can affect corrosion behavior of the zinc in concrete. Analysis was carried out using an ion chromatograph (IC), and the results are also shown in Table 1. Concrete of lower mechanical properties was chosen (w/c: 0.5; distilled water) with fine aggregates (maximal grain size: 4 mm) to ensure unlimited water transport to the surface of the high-sensitivity electrical sensor (Table 2). The mixture was compacted using a vibration table.

**Figure 3:**
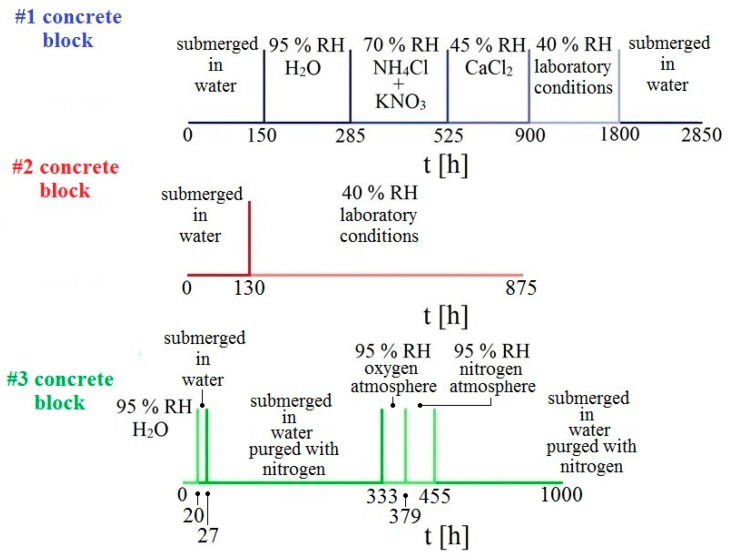
Graphical representation of experimental time schedule for individual concrete samples.

Altogether, 3 concrete samples were prepared which were then exposed in different environments. Individual samples were placed on a rack in individual containers, equipped with a rubber seal and glass desk to maintain relative humidity. The first sample was exposed in an environment with gradually decreasing humidity. The humidity was controlled by use of a suitable mixture of inorganic salts dissolved in distilled water under the rack. The second sample was exposed only in two extreme conditions, i.e., submerged underwater and at laboratory conditions of 40% relative humidity (RH). The last sample was exposed in an atmosphere deprived of oxygen (purged with gaseous nitrogen) and then in an atmosphere with an overabundance of oxygen. The overall experimental program is displayed in Figure 3. During the measurement of changes of electrical resistance of individual sensors, corrosion potential E_corr_/SCE was also measured.

**Figure 4:**
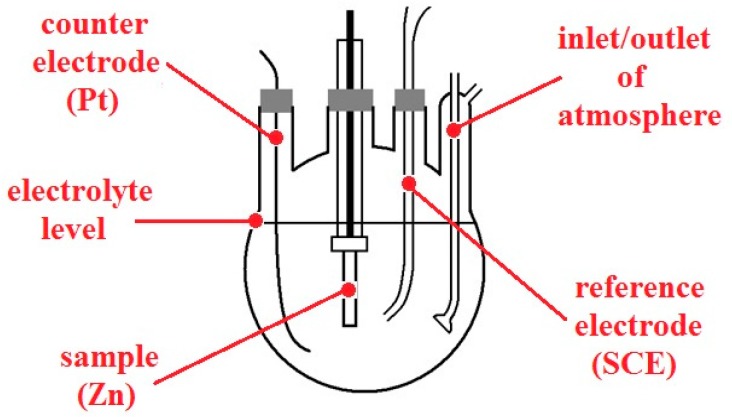
Model of three-electrode setup used for anodic polarization curve measurements.

Furthermore, anodic polarization curves of the cylindrical zinc (99.9%, area of 1280 mm^2^) sample in model pore solutions (pH 12.6 and 13.5; in the presence of Ca^2+^ and with pH adjusted with KOH) with and without oxygen (purged with nitrogen) were also measured. Nitrogen purging was started 20 min in advance and then maintained during the whole experiment. A conventional three-electrode cell with inlets for both gases was used (Figure 4), connected to recording device PC3. SCE was used as a reference electrode, with platinum wire as a counter electrode. The polarization rate was set to 0.1 mV/s. The measurements were always carried out at ambient temperature. Before every experiment, the cylindrical sample was grinded with P320 abrasive paper and degreased with acetone.

Phase composition of corrosion products precipitates on the surface of the measuring segment of the high-sensitivity zinc electrical resistance probe (embedded in concrete for #1 concrete block, 1 cm from the surface) was studied using X-ray diffraction (PANalytical, Almelo, The Netherlands), and precipitation of corrosion products on the surface of the concrete taken from the interfacial transition zone of the same concrete sample was also studied using SEM (Tescan Orsay Holding, Brno, Czech Republic).

## 3. Results and Discussion

These presented results of zinc corrosion in real concrete follow the effect of transporting oxidizing agent (water itself and dissolved oxygen) to the metallic surface, and the anti-corrosion barrier efficiency of conventional corrosion products based on Ca[Zn(OH)_3_]_2_·2H_2_O in long-term ageing conditions. Contrary to earlier research in this field, the experiments in this work are based on a direct and very accurate measurement of the corrosion rate.

**Figure 5:**
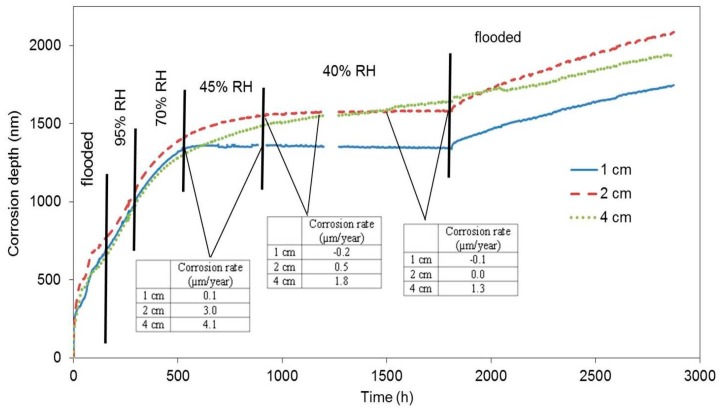
Corrosion depth records of high-sensitivity zinc electrical resistance probes embedded in concrete for #1 concrete block.

The first block was submerged in water 24 h after being cast. We assumed that the concrete pore system was permanently filled with water (maximal abundance). The response of the electrical resistance probes during the subsequent exposure is displayed as the relation between the loss of thickness of the zinc probe in nanometres and time (Figure 5). The corrosion rate and free corrosion potential values are stated in Table 3. When the block is flooded, the active zinc (free corrosion potential falls in the range between −1000 and −1100 mV vs. SCE (Saturated Calomel Electrode)) corrosion rate is very high, while hydrogen is formed [6,11,22]. Upon reducing the relative humidity to 95%, the corrosion rate dropped; however, based on the values of free corrosion potentials, zinc was still active in terms of corrosion. The corrosion rate also dropped when the relative humidity was reduced to 70%, 45%, and to laboratory humidity (40%). Upon reducing relative humidity to 45% and 40%, the highest drop in corrosion rate was recorded for the sample that had been positioned 1 cm from the surface, followed by the sample at 2 cm from the surface, and, finally, by the sample placed 4 cm from the surface (corrosion rate values in Figure 5). The variations of corrosion rate for the sample at 4 cm depth are less pronounced compared to the other sensors, probably because of a temporary persistence of conditions deeper in the concrete after rapid alternation of conditions at the concrete surface. We can thus state that the corrosion rate is dropping with reduced relative humidity. Similarly, the distance of the sample from the concrete surface plays its role as well.

Should the zinc probes lean towards passivation in concrete, a drop of corrosion rate and an increase of free corrosion potential in a positive direction would be observed in flooded concrete [5,17,18,23].

A significant drop in the corrosion rate in the case of the first block was recorded almost 22 days after the beginning of the exposure. Upon repeated flooding, the corrosion rate increased; however, the corrosion rate values were one order of magnitude lower than in the case of the freshly flooded concrete (Table 4). Nevertheless, it can be assumed, based on the value of the free corrosion potential (−1300 mV vs. SCE), that zinc remained in active state in terms of corrosion. It follows that zinc did not passivate during the time of its exposure in the concrete by the means of creating corrosion products (CHZ—calcium hydroxyzincate). The reduction of the corrosion rate is thus only probably linked to the reduced humidity, i.e., electrolyte and oxidizing agent depletion. Obviously, the barrier protection mechanism of zinc corrosion products is apparently very limited. Zinc or hot-dip galvanized steel can corrode at an unacceptable corrosion rate (more than 4 µm·a^−1^), even after 1800 h of exposure. This is, of course, incomparable with the barrier protection mechanism of steel corrosion products in concrete (without chloride ions contamination and without carbonation effect—r_corr_ < 0.1 µm·a^−1^ [2,3]).

**Figure 6:**
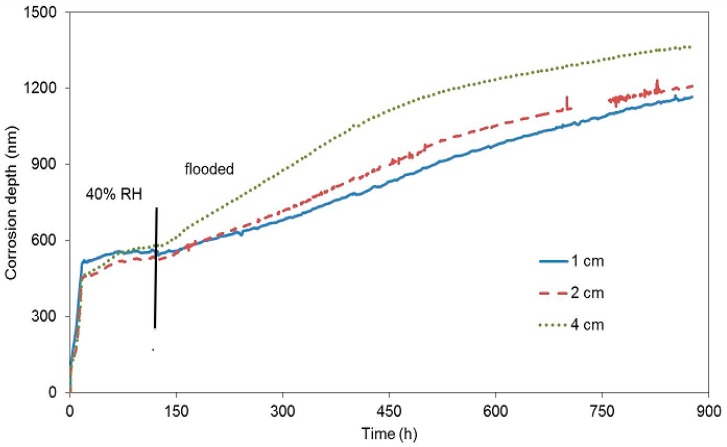
Corrosion depth records of high-sensitivity zinc electrical resistance probes embedded in concrete for #2 concrete block.

The second block was exposed to laboratory conditions where concrete was drying quickly (Figure 6), immediately after being cast. It was expected that the dropping humidity of concrete (water evaporation resulting from the drying concrete) would keep reducing the corrosion rate due to the depletion of the oxidizing agent. The corrosion rate in the beginning of the exposure was one order of magnitude higher than it was in the case of the first block. This fact is probably due to an easier access of oxygen in gaseous form through the drying pores under common laboratory conditions. A decrease of the corrosion rate was recorded after approximately 17 h, when the free corrosion potential began to grow significantly. This response was probably caused by reduced humidity from the drying concrete. After six days, the concrete block was submerged in water, making sure humidity entered the concrete through a single surface facing the surface of the zinc electrical resistance probes. Even in this case, the corrosion rate grew and the free corrosion potential dropped towards negative values (Table 5). The change of free corrosion potential to −1300 mV vs. SCE shows that water reduction was the dominant cathodic reaction after flooding. The change of the slope of the curves in Figure 6 after immersion clearly shows a change in corrosion rate of all the embedded probes. Neither of the experiments with concrete blocks demonstrated the creation of a protective passive layer formed by corrosion products.

**Figure 7:**
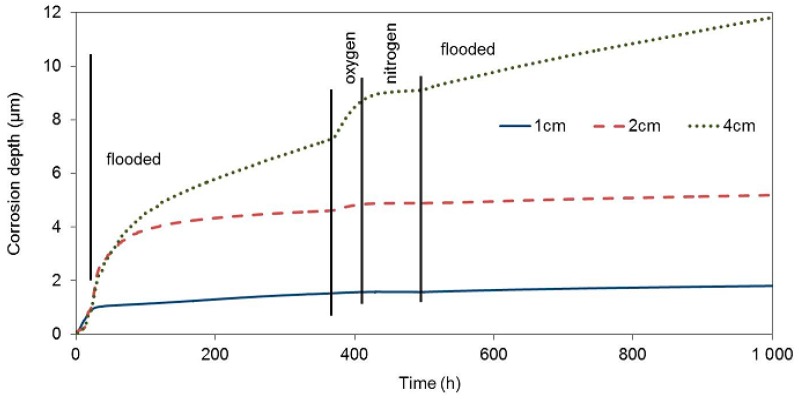
Corrosion depth records of high-sensitivity zinc electrical resistance probes embedded in concrete for #3 concrete block.

The test of the impact of corrosion environment oxygenation on the zinc corrosion behaviour in concrete took place under the same experimental conditions as the tests of the impacts of the presence of water. Upon being cast, the third concrete block with electrical resistance probes was placed in an atmosphere with a relative humidity of 95%, which prevents concrete from drying. On the other hand, the surrounding atmosphere has easier access to the concrete pore system in comparison with the situation where the concrete is fully flooded. The corrosion rate of all three electrical resistance probes was similar during this entire phase (20 h), and it corresponded to the corrosion rate in drying concrete (Figure 7, Table 6).

During the next phase, when the concrete block was exposed in deoxygenated water (27th through 360th hour), the corrosion rate of the zinc sensor closest to the block surface essentially decreased immediately. The free corrosion potential in flooded concrete was at the thermodynamic water stability region, which is positive to −1038 mV in an environment with a pH of 13.5, with respect to the saturated calomel electrode. The corrosion rate of this sensor was probably significantly increased prior to deoxygenating the water bath, due to the reduction of the present oxygen. Upon eliminating this component of the cathodic process, zinc continued to corrode in the active state, which was demonstrated by a significantly negative value of the free corrosion potential, although with a significantly lower corrosion rate. The other two probes, inserted deeper, continued to corrode at a high corrosion rate. The corrosion rate slowed down earlier for the sensor located at 2 cm. Residual oxygen dissolved in the pore water probably played its role here. 

After 360 h of exposure, the concrete block was moved to a box with an oxygen atmosphere. After approximately 10 hours of exposure in the oxygen atmosphere, the corrosion rate of the zinc probe located 4 cm and 2 cm under the concrete surface significantly increased. No corrosion rate change was recorded for the probe at 1 cm, despite the fact that we expected this probe to start corroding faster the earliest, due to the increased content of oxygen. This fact could be explained by the transfer of zinc to passivity in the environment with a high oxidizing power. The free corrosion potentials of all three probes in the oxygen atmosphere environment moved to the water thermodynamic stability region. The corrosion rate was thus not affected by water reduction on the zinc surface anymore. When oxygen was exchanged for nitrogen, the corrosion rate decreased to very low values. The contribution of water reduction is seen again after flooding the concrete block with deoxygenated water when the corrosion rate of the probes in 1 and 2 cm depth increased by an order of magnitude. 

**Figure 8:**
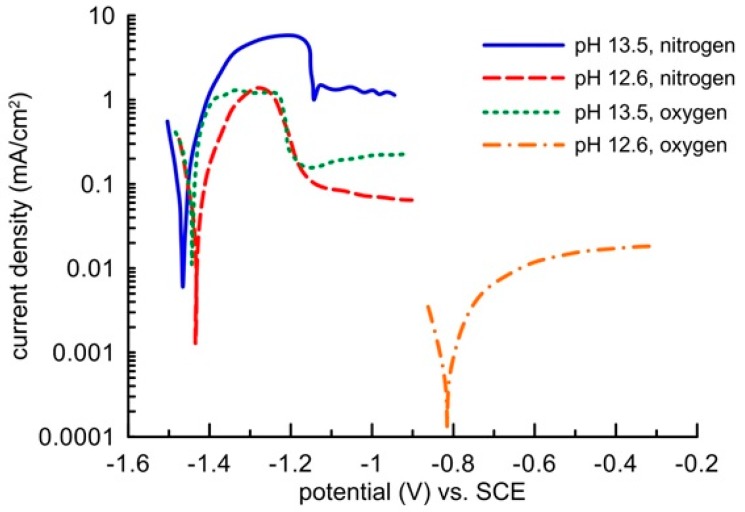
Potentiodynamic curves of zinc in oxygenated or deoxygenated model concrete pore solutions of pH 12.6 or 13.5 (scanning rate 1 mV/s)**.**

Zinc passivation in an environment with a high oxidizing power given by oxygen dissolved from the oxygen atmosphere was demonstrated by electrochemical measurements in a model concrete pore solution (Figure 8). In a saturated Ca(OH)_2_ solution, the pH of which was modified to 13.5, the sample showed the same free corrosion potential in the region of thermodynamic hydrogen stability (out of the water stability region) independently of the environment’s oxidizing power. The corrosion rate was identical in the deoxygenated as well as oxygenated solution. It amounted to approximately 0.1 mA/cm^2^. The corrosion reaction was thus predominantly controlled by water reduction, and the increased content of oxygen had no impact on the corrosion rate of the clean zinc surface. Blockage of the zinc surface by a layer of corrosion products appeared on the anodic polarization curve only after increasing the zinc potential to more positive values above −1150 mV (SCE), when zinc showed signs of transfer from activity to passivity (but very low barrier protection ability). Formation of this layer was initiated unnaturally by a high passing current from an external source, which probably resulted in electrolyte oversaturation by zinc corrosion products, similarly to a drying concrete. The precipitation of the layer was probably also helped by acidification of the solution along the surface of the working electrode, which undoubtedly occurs on the anodically polarized sample when the impressed current passes through. This claim was confirmed by the shape of the polarization curves, which were recorded in the Ca(OH)_2_ saturated solution without any further modification of pH, i.e., at a pH of 12.6. Zinc in the electrolyte without oxygen remained in an active state and, similarly to the environment with a pH of 13.5, the corrosion rate dropped upon anodic polarization. When the electrolyte with a pH of 12.6 was being saturated with oxygen, zinc spontaneously passed to the passivity area, and the barrier quality was undoubtedly greater than that with a pH of 13.5. The corrosion rate was up to two orders of magnitude lower (Figure 8). The zinc corrosion behaviour in the alkali environment of concrete thus strongly depends not only on the presence of water as an oxidizing agent, but also on the presence of oxygen.

**Figure 9:**
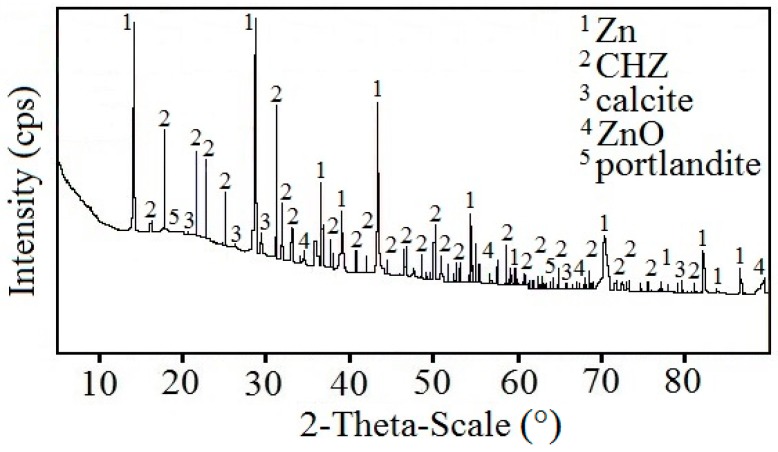
Diffractogram of precipitated corrosion products on the surface of the sensing part of the high-sensitivity zinc electrical resistance probes embedded in concrete for #1 concrete block (10 mm from the surface).

**Figure 10:**
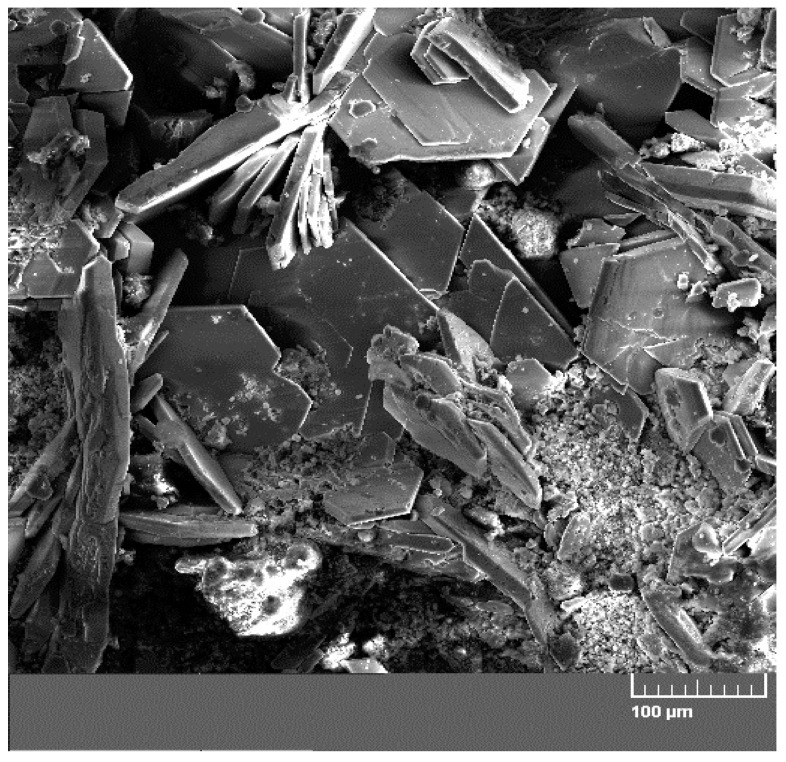
SEM image of concrete from the phase interphase of the sensing part of the high-sensitivity zinc electrical resistance probes (#1 concrete block—10 mm from the surface) with vertical plate crystallites of calcium hydroxyzincate (CHZ) (horizontal crystallites are portlandite—Ca(OH)_2_).

Figure 9 displays a diffractogram of phases precipitated on the sensing part of the high-sensitivity zinc electrical resistance probe. The major constituent of the corrosion products is Ca[Zn(OH)_3_]_2_·2H_2_O (CHZ—calcium hydroxyzincate) which, according to the literature, corresponds to the development of salt passivity on the hot-dip galvanized rebar in concrete [5,11,14,24]. Results of this experiment disprove this, since the sensing part of the probe provably did not undergo the transition to passive state even at 1800 h after the start of the exposure in real concrete. The ratio of ZnO in corrosion products is negligible. This kind of experiment can also confirm the negative effect of Ca^2+^ on the evolution of the passive layer on zinc or galvanized rebar in model concrete pore solution [16,18] or in real concrete [17]. SEM images also confirm the presence of CHZ (Figure 10) [11,25].

The effect of water transport on zinc corrosion in concrete has already been indicated in previous research [26]. The limited anti-corrosion properties of Ca[Zn(OH)_3_]_2_·2H_2_O-based corrosion products have also been described previously [27].

## 4. Conclusion

This paper studies in detail the corrosion behavior of zinc in concrete using high-sensitivity zinc electrical resistance probes to directly determine changes in corrosion rate as well as E_corr_. The effect of water presence (by varying relative humidity of the ambient atmosphere) and excess of oxygen were the main areas of focus.

Based on the long-term exposures (#1 concrete block up to 2850 h), the widely accepted theory of easy passivation of zinc on hot-dip galvanized rebar can be decisively disproven, which ties to the formation of typical crystalline corrosion products in this environment—Ca[Zn(OH)_3_]_2_·2H_2_O (CHZ—calcium hydroxyzincate). The barrier mechanism of protection of the zinc surface (or hot-dip galvanized steel) in concrete by this corrosion product is apparent from our data; however, its effect is very limited. Corrosion of zinc and galvanized rebar in concrete is, even during long exposures, facilitated mainly by water as a depolarization agent in cathodic corrosion reaction. Elevated oxygen concentration supports corrosion of zinc in concrete; however, it can also lead to passivation of the zinc surface (it is still strongly dependent on the pH of the environment). This supports modern theory—the passivation of zinc surface (or galvanized rebar) due to the presence of ZnO and Zn(OH)_2_ in the pores between crystallites of Ca[Zn(OH)_3_]_2_·2H_2_O. These corrosion products form more easily in the presence of higher concentrations of oxygen. 

It is apparent from the practical standpoint that the corrosion rate of galvanized rebar is highly dependent on the access of water to the surface of the rebar. When passivation of the surface by the zinc corrosion products is excluded, zinc corrosion can lead to unacceptable thinning of the rebar, significantly reducing bond strength between rebar and concrete. In the case of using the galvanized rebar, it is necessary to consider using additional protection of the rebar by top coating (e.g., conversion coating).

## Figures and Tables

**Table 1:** Cement composition guaranteed by producer—general and minorities (%).

Compound	CaO	SiO_2_	Al_2_O_3_	Fe_2_O_3_	SO_3_	MgO	Na_2_O	K_2_O	Cl^−^	CrO_4_^2−^
Content, %	57.5	22.3	4.6	5.4	3.5	1.2	0.11	0.97	3.1 × 10^−5^	~2.0 × 10^−5^

**Table 2:** Characteristic information about commercial concrete mixture guaranteed by the producer.

Concrete Grade	Maximal Grain Size (mm)	Mixture	Cubic Compression Strength after 28 Days of Curing (MPa)
C16/20	4	w/c: 0.50	>20

**Table 3:** Free corrosion potential and corrosion rate of electrical resistance probes embedded in concrete at various distances from the concrete surface, which was exposed to varying conditions in terms of humidity—starting with a period of immersion in water.

Distance	Flooded		95% RH		70% RH
	Corrosion Rate (µm/year)	Free Corrosion Potential (mV) vs. SCE	Corrosion Rate (µm/year)	Free Corrosion Potential (mV) vs. SCE	Corrosion Rate (µm/year)
1 cm	28.2	−936	19.0	−1054	13.8
2 cm	29.6	−1107	17.4	−1132	13.9
4 cm	24.6	−1136	20.2	−1098	13.2

**Table 4:** Free corrosion potential and corrosion rate of electrical resistance probes embedded in concrete at various distance from the concrete surface that was exposed to varying conditions in terms of humidity—continuation of Table 3.

Distance	45% RH	40% RH		Flooded	
	Corrosion Rate (µm/year)	Corrosion Rate (µm/year)	Free Corrosion Potential (mV) vs. SCE	Corrosion Rate (µm/year)	Free Corrosion Potential (mV) vs. SCE
1 cm	0.1	−0.2	−625	3.2	−1329
2 cm	3.0	0.2	−633	4.1	−1322
4 cm	4.1	1.5	−702	2.2	−1302

**Table 5:** Free corrosion potential and corrosion rate of electrical resistance probes embedded in concrete at various distances from the concrete surface, which was exposed to varying conditions in terms of humidity—starting with period of drying in air with 40% relative humidity.

Distance	40% RH	40% RH		Flooded	
	Corrosion Rate (µm/year) 1–17.25 h	Corrosion Rate (µm/year) 17.5–145.25 h	Free Corrosion Potential (mV) vs. SCE	Corrosion Rate (µm/year)	Free Corrosion Potential (mV) vs. SCE
1 cm	233.1	2.5	−483	7.7	−1227
2 cm	199.5	5.7	−477	8.3	−1271
4 cm	188.7	9.3	−488	8.9	−1296

**Table 6:** Free corrosion potential and corrosion rate of electrical resistance probes embedded in concrete at various distances from the concrete surface, which was exposed to varying conditions in terms of humidity and oxidizing power of the environment.

Distance from the Surface:		1 cm	2 cm	4 cm
Humid air	Corrosion rate (µm/year)	189	406	368
	Free corrosion potential (mV) vs. SCE	−917	−895	−820
Flooded	Corrosion rate (µm/year)	181	1227	1116
	Free corrosion potential (mV) vs. SCE	−1060	−1085	−1085
Flooded + N_2_	Corrosion rate (µm/year)	9	58	132
	Free corrosion potential (mV) vs. SCE	−1042	−1025	−1022
Oxygen	Corrosion rate (µm/year)	8	29	216
	Free corrosion potential (mV) vs. SCE	−360	−470	−515
Nitrogen	Corrosion rate (µm/year)	0.7	0.8	26
	Free corrosion potential (mV) vs. SCE	−525	−500	−553
Flooded + N_2_	Corrosion rate (µm/year)	9	3	59
	Free corrosion potential (mV) vs. SCE	−770	−750	−760
